# A Rare Case of Vancomycin-Induced Immune Thrombocytopenia

**DOI:** 10.7759/cureus.23328

**Published:** 2022-03-19

**Authors:** Maha Hameed, Sultan Alamri, Sami Almustanyir

**Affiliations:** 1 College of Medicine, Alfaisal University, Riyadh, SAU; 2 Critical Care Department, National Care Hospital, Riyadh, SAU; 3 Internal Medicine, Ministry of Health, Riyadh, SAU

**Keywords:** heparin, ditp, thrombocytopenia, drug-induced thrombocytopenia, vancomycin

## Abstract

Vancomycin-induced immune thrombocytopenia (ITP) is a rare type of drug-induced immune thrombocytopenia (DITP) that can lead to life-threatening consequences as a result of its use. We herein report a case of a 74-year-old male with a history of diabetes mellitus type II, Alzheimer’s disease, and stroke who presented to the ICU with sepsis. The patient was on heparin for 20 days prior to presentation, with platelet levels at the time within normal limits as per his baseline. Following the introduction of vancomycin to his clinical regimen in the treatment of sepsis, the patient developed a significant drop in platelet count from 400 x10³/mm³ to 70 x10³/mm³. Discontinuation of the drug leads to improvement of the platelet counts confirming the diagnosis of vancomycin-induced ITP.

## Introduction

More than 300 drugs have been historically implicated in the development of drug-induced immune thrombocytopenia (DITP), a potentially lethal clinical syndrome due to the difficulty in diagnosis and under-recognition by clinicians [1]. Commonly implicated in the development of this condition include hematologic (e.g., abciximab, eptifibatide, heparin, tirofiban, oxaliplatin), cardiologic (e.g., quinidine), and neurologic (e.g., carbamazepine, mirtazapine) medications. Nonsteroidal anti-inflammatory drugs (NSAIDs) such as ibuprofen, and antimicrobial agents (e.g., ceftriaxone, penicillin, quinine, rifampicin, trimethoprim-sulfamethoxazole, and vancomycin) have also been widely explored in literature [1-2].

Various mechanisms have been implicated in the development of DITP, the most common being the production of platelet antibodies in response to the drug, leading to platelet destruction due to the reticuloendothelial system. This can result in severe thrombocytopenia that can further manifest in the form of petechiae, ecchymosis, and mucosal bleeding (e.g., epistaxis, hematuria). However, as this drug-dependent thrombocytopenia is primarily immune-mediated, platelet counts typically return to baseline once discontinuation of the medication has been established [3].

While the first case of vancomycin-induced thrombocytopenia has been reported as early as 1985 [4], there is still a notable lack of literature documenting this rare side effect. We herein report a case of vancomycin-induced ITP diagnosed by clinical evidence of platelet count improvement upon discontinuation of the drug.

## Case presentation

A 74-year-old male with diabetes mellitus type II, Alzheimer's disease, and a previous history of stroke was transferred to the ICU from the chronic care ward due to sepsis from infected sacral bed sores. On examination, the patient was pale, cachectic, tachycardiac (heart rate of 120 beats per minute), tachypneic (respiratory rate of 28 breaths per minute), hypotensive (blood pressure of 100/70), increased capillary refill time of 3 seconds, and O2 saturation of 84% on 3 liters of oxygen via nasal cannula. In addition, decreased bilateral air entry was noted on chest examination, and the abdomen was soft, lax, with suprapubic tenderness.

Twenty days before presentation, the patient was started on heparin for pulmonary embolism prevention (due to high-risk as per the patient's prolonged history of immobilization). The patient's medications consisted of only aspirin for secondary stroke prophylaxis and insulin for diabetes management. His platelet counts were within normal limits during the entire length of his hospital course before sepsis development. A complete blood count (CBC) done upon admission to the ICU revealed a high WBC count (11,000/mm³), low hemoglobin (8 g/dL), and a normal platelet level of 400 x10³/mm³. Serum electrolytes were only remarkable for hypokalemia (2.8 mEq/L) and elevated C-reactive protein (100 mg/L). Renal function and liver function tests were within normal limits.

**Figure 1:**
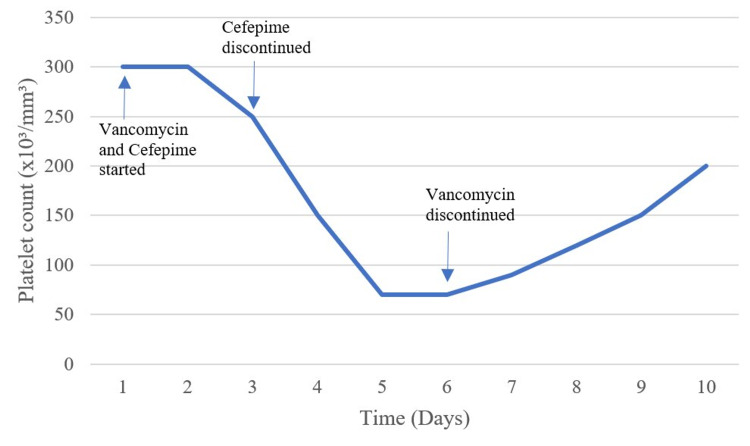
Platelet count in relation to vancomycin use.

He was immediately started on an empirical regimen of IV vancomycin 1 gm every 12 hours and IV cefepime 2 gm every eight hours. On day 3 of empirical management, cefepime was discontinued as per the patient's blood and skin culture results. By day 5 of vancomycin use, the patient had a drop in platelets to 70 x10³/mm³ (Figure 1). Vancomycin was then discontinued on day 6 due to concerns of vancomycin-induced thrombocytopenia. The patient's platelet levels went back to baseline upon subsequent platelet count checks. Follow-up labs conducted later when the patient was transferred back to the chronic ward revealed similar baseline measurements.

## Discussion

Vancomycin is a glycopeptide antibiotic derived from Streptococcus orientalis and is used to treat infections caused by Gram-positive bacteria, methicillin-resistant Staphylococcus aureus (MRSA), streptococci, and enterococci. Its mechanism of action involves inhibiting the polymerization of peptidoglycans in the cell wall of bacteria, thereby causing a bactericidal effect due to the leakage of intracellular contents through the weakened cell walls [5].

Common side effects noted with IV vancomycin as used in our patient include nephrotoxicity, hypotension, anaphylaxis, and red man syndrome. Other side effects are less common, such as local phlebitis, drug-induced fever, skin rash, chills, eosinophilia, and neutropenia. Rarer consequences include drug rash with eosinophilia and systemic symptoms (i.e., DRESS syndrome), ototoxicity, vasculitis, Stevens-Johnson syndrome, and thrombocytopenia which is noted in our patient [5].

The mechanism by which vancomycin has been proposed to cause thrombocytopenia involves a suspected proapoptotic antibody-independent effect through Ca2+ signaling, mitochondrial depolarization, and phosphatidylserine exposure in the platelet membranes [1]. Heparin-induced thrombocytopenia (HIT) has been extensively studied, in contrast to vancomycin-induced thrombocytopenia due to a higher incidence of thrombocytopenia in 10-30% of patients treated with heparin vs. less than 1% seen with vancomycin use [6].

HIT is subdivided into nonimmune heparin-associated thrombocytopenia (HIT type 1) and immune-mediated HIT (HIT type 2). The pathophysiology of both types involves the production of antibodies against platelet factor 4 (PF4)/heparin complexes. HIT type 1 usually occurs within five days of heparin therapy and is typically mild with rare clinical consequences. However, HIT type 2 develops between five to ten days of heparin therapy and can occur in the presence of a thromboembolic event [6]. 

Although our patient has a history of heparin use before presentation, the development of thrombocytopenia 20 days after heparin initiation falls out of the clinical time course of both HIT type 1 and type 2. In addition, the resolution of thrombocytopenia following the discontinuation of vancomycin further confirms the diagnosis of vancomycin-induced immune thrombocytopenia.

Additional studies to evaluate for differential diagnoses such as thrombotic thrombocytopenic purpura, disseminated intravascular coagulopathy, atypical hemolytic uremic syndrome were not done due to the clear role of vancomycin being the direct cause of the thrombocytopenia seen in our patient soon after its commencement. In addition, flow cytometry to test for the presence of IgM and IgG antibodies to vancomycin was similarly not conducted in our patient due to the lack of ambiguity in determining the etiology of the thrombocytopenia.

## Conclusions

This case identifies the importance of the clinical recognition of vancomycin-induced ITP. Typically, a commonly overlooked condition, vancomycin-induced ITP is rarely reported in the literature due to the difficulty in determining the causative agent in a patient on multiple medications. However, given the severe consequences, including the risk of morbidity and mortality, further awareness regarding this presentation is warranted.

## References

[1] Bakchoul T, Marini I (2018). Drug-associated thrombocytopenia. Hematology Am Soc Hematol Educ Program.

[2] Arnold DM, Nazi I, Warkentin TE, Smith JW, Toltl LJ, George JN, Kelton JG (2013). Approach to the diagnosis and management of drug-induced immune thrombocytopenia. Transfus Med Rev.

[3] MacDougall KN, Parylo S, Sokoloff A (2020). A case of vancomycin-induced immune thrombocytopenia. Cureus.

[4] Walker RW, Heaton A (1985). Thrombocytopenia due to vancomycin. Lancet.

[5] Patel S, Preuss CV, Bernice F (2022). Vancomycin. https://www.ncbi.nlm.nih.gov/books/NBK459263/.

[6] Von Drygalski A, Curtis BR, Bougie DW (2007). Vancomycin-induced immune thrombocytopenia. N Engl J Med.

